# How Can Biological and Chemical Silver Nanoparticles Positively Impact Physio-Chemical and Chloroplast Ultrastructural Characteristics of *Vicia faba* Seedlings?

**DOI:** 10.3390/plants12132509

**Published:** 2023-06-30

**Authors:** Bushra Ahmed Alhammad, Heba M. M. Abdel-Aziz, Mahmoud F. Seleiman, Shaimaa M. N. Tourky

**Affiliations:** 1 Biology Department, College of Science and Humanity Studies, Prince Sattam Bin Abdulaziz University, Al Kharj Box 292, Riyadh 11942, Saudi Arabia; 2 Botany Department, Faculty of Science, Mansoura University, Mansoura 35516, Egypt; 3 Plant Production Department, College of Food and Agriculture Sciences, King Saud University, P.O. Box 2460, Riyadh 11451, Saudi Arabia; 4 Department of Crop Sciences, Faculty of Agriculture, Menoufia University, Shibin El-Kom 32514, Egypt

**Keywords:** AgNPs, *Vicia faba*, *Jatropha curcas*, growth traits, chloroplast, antioxidant systems

## Abstract

Through interactions with plant cells, silver nanoparticles (AgNPs) with both biological and chemical origins can stimulate physiological and metabolic processes in plants. To ensure their safe application in the food chain, it is necessary to investigate their effects on plant systems. Therefore, the effects of chemical AgNPs (chem-AgNPs) and biologically synthesized AgNPs (bio-AgNPs) at different levels (i.e., 0, 10, and 50 ppm) on physiological and biochemical traits {i.e., root and shoot growth traits, photosynthetic pigments (Chl *a*, Chl *b*, carotenoids, and total pigments), soluble sugars, total carbohydrates, starch, H_2_O_2_, and antioxidant enzyme activities} of *Vicia faba* L. seedlings were investigated. AgNPs were biosynthesized from silver nitrate (AgNO_3_) by a green synthesis approach using *Jatropha curcas* seed extract. The synthesized AgNPs were characterized by UV-vis spectroscopy, transmission electron microscopy (TEM), zeta potential, Fourier-transform infrared spectra (FT-IR), and X-ray diffraction (XRD). The results showed that bio-AgNPs at 10 ppm resulted in the highest growth, physiological, and biological traits of faba bean seedlings in comparison with those obtained from both AgNO_3_ and chem-AgNPs treatments. On the other hand, all AgNPs treatments adversely affected the chloroplast ultrastructure, however, fewer negative effects were obtained with the application of 10 ppm bio-AgNPs. In addition, the roots and shoots of seedlings contained the lowest Ag content under different treatments at 10 ppm AgNPs in comparison to the highest level of AgNPs (50 ppm), which indicates that additional studies should be incorporated to ensure safe use of lower concentrations of bio-AgNPs in seed priming. In conclusion, the application of biogenic nanoparticles at 10 ppm can be recommended to enhance plant growth and the productivity of strategic crops.

## 1. Introduction

Faba bean (*Vicia faba*) is one of the most important crops, particularly among legumes, because it contains different organic compounds as well as high percentages of protein and minerals [1,2]. Moreover, it can enhance nitrogen fixation in the soil and consequently can improve its fertility [2,3]. Therefore, enhancing growth and productivity of faba bean by using new approaches such as NPs is considered an important issue.

Seed priming is a pre-sowing process that coats seeds with specific compounds at the ideal concentration for a certain period of time under controlled conditions to improve seed germination and physiological traits of crops grown under abiotic stress [4,5,6]. In addition, nanomaterials (NMs) can promote seed germination, plant growth, and productivity of plants grown under different environmental stresses, particularly when applied as seed priming [7,8]. Silver nanoparticles (AgNPs) are considered the most commonly generated engineered NPs, and are used in a wide range of agro-commercial products [9,10].

There are several applications for AgNPs in biological systems. According to nano-research data, AgNPs are a broad-spectrum nanomaterial for use in agriculture. However, nanotoxicological studies have revealed that AgNPs have a size- and concentration-dependent impact on biological systems. In practice, these NPs are a double-edged sword [11]. Numerous studies have examined the positive and negative impacts of AgNPs on the progression of plant growth indices. Regarding positive impacts, the influence of AgNPs on improving seedlings and growth of the plant shoot and root as well as the use of AgNPs for seed priming to speed up germination have been investigated [12,13]. Furthermore, two cultivars of *Phaseolus vulgaris* exposed to low levels of AgNPs increased their seed germination as well as their physiological traits [14]. Nanoparticles including AgNPs have shown beneficial effects on different crops grown under different abiotic and biotic stresses [15,16]. Seed germination was positively affected by treatment with AgNPs in *Boswellia ovalifoliolata* plants and in *Pennisetum glaucum* [17,18]. Regarding negative impact, the behavior of AgNPs in relation to plant/seedlings has been studied in a number of studies [19,20].

AgNPs can be synthesized through physical, chemical, or biological synthesis; however, biological synthesis has emerged as a viable alternative owing to its low cost and environmentally acceptable manufacturing techniques. Despite a paucity of knowledge on their toxicological effects, particularly on plant–soil systems, biosynthesized AgNPs have been extensively used in a variety of applications in recent years [21]. Since it does not require the time-consuming process of maintaining cell cultures, the use of plant extract to produce nanoparticles may be better than other environmentally friendly biological procedures [22,23]. Green metal nanoparticles production is effectively used in the synthetic method that employs biomass or plant extracts. Green AgNPs have recently been synthesized using natural products such as green tea (*Camellia sinensis*) [24], neem (*Azadirachta indica*) leaf broth [25], natural rubber [26], *Aloe vera* plant extract [27], leguminous shrub (*Sesbania drummondii*) [28], and *Jatropha curcas* latex [29]. 

*Jatropha curcas* is an economically valuable tree. It has been recognized as a possible biodiesel crop due to the presence of 40–50% oil in the seed, which can be transformed into biodiesel via a chemical or lipid-mediated esterification process [30]. The kernel of the Jatropha seed yields 40–60% oil as a valuable commodity. During their development processes, biomolecules containing carbonyl, hydroxyl, and amine functional groups have the capacity to reduce metal ions and cap newly generated particles [31]. *Jatropha curcas* latex may be utilized to reduce Ag^+^ to Ag, and latex components can also function as a capping agent to stabilize Ag nanoparticles [29].

Because of the dynamic nature of AgNPs’ absorption, translocation, accumulation, and destiny, as well as their subsequent dual impacts within various plant species, there is no agreement on the overall influence of AgNPs on plant morphology, physiology, and metabolism. This has motivated more mechanistic research [29]. Furthermore, if the amount of toxicity created by biogenic AgNPs is lower in plant systems, they may be an effective alternative for chemically produced AgNPs in a number of relevant goods. As a result, more research is needed to uncover thoroughly the mechanisms influencing physiological and biochemical processes in AgNPs-treated plants, as well as their stimulation or inhibition, and to reconcile these seemingly contradictory results.

Therefore, the present study aimed to (1) investigate the preparation and characterization of chemically (chem-AgNPs) and biologically (bio-AgNPs) synthesized silver nanoparticles from *Jatropha curcas* seeds in terms of morphology (size and shape), TEM (transmission electron microscopy), zeta potential, FT-IR, and XRD, and to (2) investigate the effects of biogenic AgNPs in comparison with chemical AgNPs on germination traits (i.e., root and shoot vigor index), physio-chemical indices (i.e., photosynthetic pigments, soluble sugars, total carbohydrates, and starch(, oxidative stress traits (i.e., H_2_O_2_ content, CAT, POX, and PPO activities), and changes in the ultrastructure of chloroplasts and accumulation of Ag in shoots and roots of faba bean (*Vicia faba* L.) seedlings.

## 2. Results

### 2.1. Characterization of the Prepared Chem-AgNPs and Bio-AgNPs

**Figure 1:**
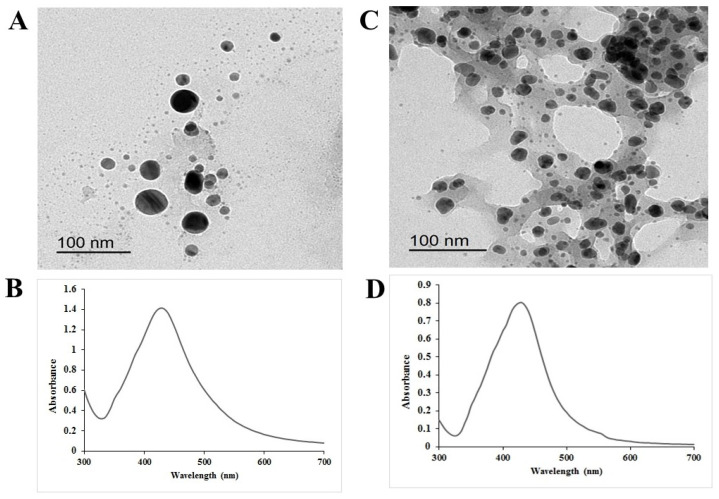
Characterization of chemical (**A**,**B**) and biological (**C**,**D**) silver nanoparticles. (**A**,**C**) Spherical shaped nanoparticles by TEM, and (**B**,**D**) UV-VIS spectrum showing peak at 430 nm.

Figure 1 reveals that the obtained chem-AgNPs appeared spherical in shape and showed an average mean diameter of 40 ± 2 nm. Meanwhile, bio-AgNPs also appeared spherical in shape with a smaller mean diameter of about 20 ± 2 nm. The average zeta potential obtained for chemically synthesized silver nanoparticles was −40.03 mV. On the other hand, the average zeta potential obtained for biologically synthesized silver nanoparticles was −22.63 mV. The UV-VIS spectroscopic observation of both AgNPs showed a peak at 430 nm. The FT-IR spectra of both AgNPs are shown in Figures S1 and S2. FT-IR analysis confirmed the chemical synthesis of silver nanoparticles (Figure S1). The carbonyl group of the carboxylic function of citrate was indicated by the presence of sharp broad absorption at 1612 and 1387 cm^−1^. The spectrum showed the presence of other absorptions at 1278, 1192, 1079, 843, and 754 cm^−1^, which indicated the formation of citric acid (indicating the use of trisodium citrate in the synthesis reaction) and the presence of other functional groups. FT-IR spectroscopy measurements were carried out to identify the formation of biologically synthesized silver nanoparticles (Figure S2). Figure S2 shows the presence of three bands at 1763, 1652, and 1386 cm^−1^. The strong absorption at 1763 cm^−1^ is due to the carbonyl stretching vibration of the acid groups of different fatty acids present in the extract. The band at 1652 cm^−1^ is characteristic of the amide I band. The band at 1386 cm^−1^ is assigned to the methylene scissoring vibrations from the proteins.

**Figure 2:**
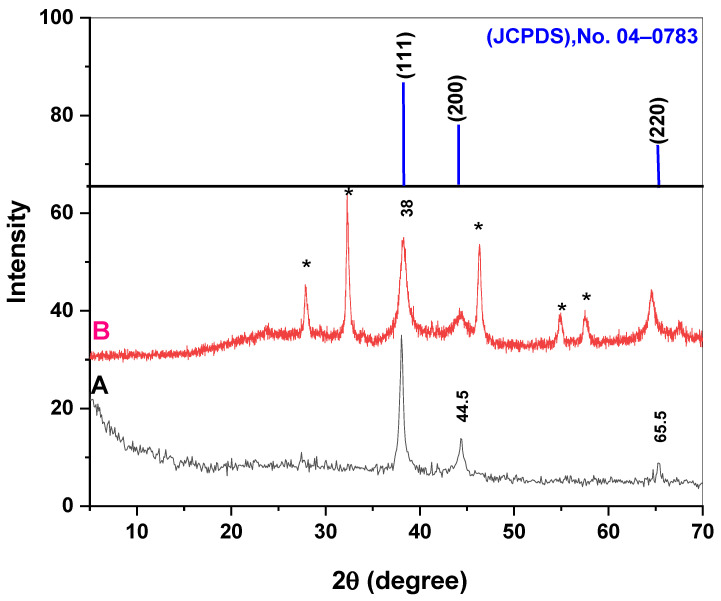
X-ray diffraction (XRD) pattern of (A) chemically synthesized Ag nanoparticles (Chem-AgNPs) and (B) biologically synthesized Ag nanoparticles (Bio-AgNPs). Peaks with asterisks are characteristic of AgCl NPs.

Figure 2 shows the XRD pattern for both types of AgNPs. A number of Bragg reflections with 2θ values of 38°, 44.5°, and 56.5° corresponding to the (111), (200), and (220) sets of the lattice planes were clearly observed, which can be indexed as the bands for face-centered cubic structures of silver in both types of AgNPs. It was also observed that the XRD pattern for AgCl NPs (with 2θ values of 27.88°, 32.26°, 46.25°, 54.85°, 57.50°) was present in the bio-AgNPs (shown with an asterisk above the peaks). The particle size of Ag and AgCl was obtained by the Scherrer equation. The values (111) of Ag and (200) of AgCl were selected to obtain the particle size. The particle size was estimated at 36.90 (d = 23.32 nm) and 62.63 (d = 20.33 nm) nm for Ag and AgCl, respectively, in bio-AgNPs (Figure 2). Meanwhile, when (111) of Ag was chosen to obtain the particle size of chem-AgNPs, the particle size was estimated at 68.90 (d = 23.32 nm) nm. XRD data were also used to calculate the weight and volume percentages of the phases present in the nanoparticles in bio-AgNPs. The weight percentages were 31.54 and 68.46% for Ag and AgCl, respectively (Figure 2). The volume percentages were 47.72 and 52.28% for Ag and AgCl, respectively (Figure 2). It is apparent that the particle size calculated from the XRD data was less than that obtained from TEM microphotographs.

### 2.2. Changes in Growth Vigor of Root and Shoot 

**Figure 3:**
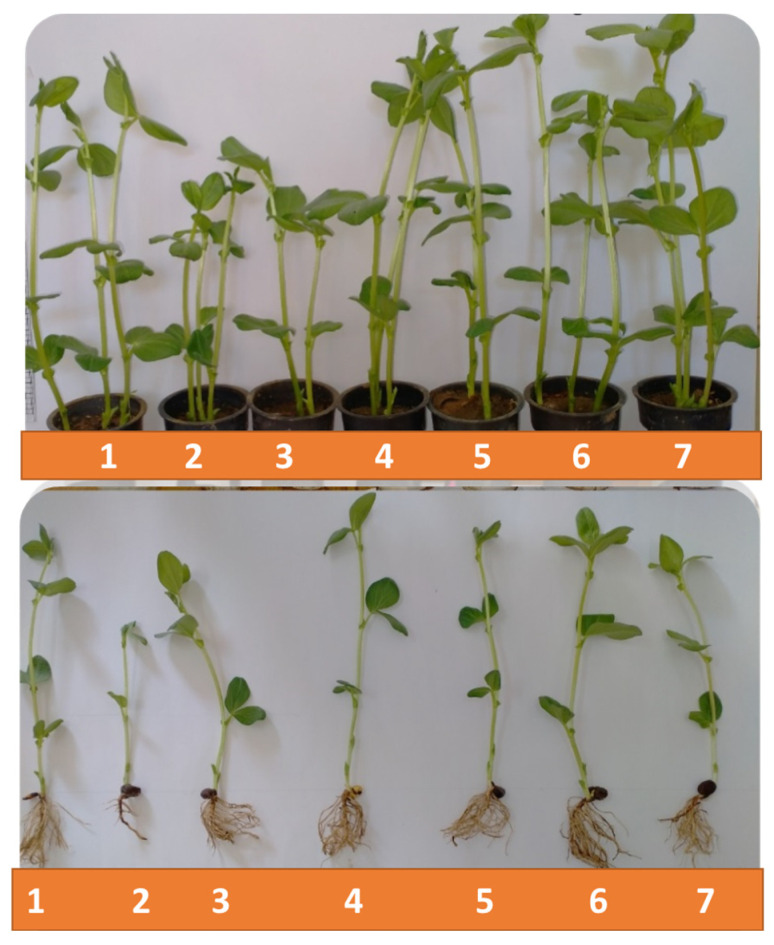
Growth of faba bean plants under different treatments of chemical and biological AgNPs at 21 DAS. Treatments are as follows: (1) control, (2) 10 ppm AgNO_3_, (3) 50 ppm AgNO_3_, (4) 10 ppm chemical AgNPs, (5) 50 ppm chemical AgNPs, (6) 10 ppm biological AgNPs, and (7) 50 ppm biological AgNPs.

The influence of priming in AgNO_3_ and AgNPs (chem- and bio-) at concentrations of 10 and 50 ppm on root and shoot vigor of faba bean seedlings are shown in Table 1 and illustrated in Figure 3. The AgNO_3_ concentrations (10 and 50 ppm) showed a decrease in all values of root growth vigor, which was significant for fresh weight and water content, and non-significant (*p* ≤ 0.05) for length and dry weight, compared with the control value (Table 1). For AgNPs (chem- and bio-) treatments, root length was the only parameter that showed a variable increase with both concentrations (10 and 50 ppm), which was significant (*p* ≤ 0.05) with bio-AgNPs treatment at a concentration of 10 ppm, compared with the control. Fresh and dry root weight and water content showed a non-significant (*p* ≤ 0.05) decrease with both types of AgNPs treatments, except for an increase recorded with bio-AgNPs treatment at a concentration of 10 ppm which was significant (*p* ≤ 0.05) for root dry weight, compared with the control (Table 1).

For shoot growth vigor, AgNO_3_ at concentrations of 10 and 50 ppm showed a non-significant (*p* ≤ 0.05) decrease in all shoot parameters (except shoot length, number of leaves, and shoot dry weight only at 10 ppm concentration) that recorded a significant decrease (*p* ≤ 0.05), compared with the control (Table 1). On the other hand, all AgNPs concentrations showed a non-significant (*p* ≤ 0.05) increase in shoot fresh weight and shoot water content, while shoot dry weight, number of leaves, and total leaf area were associated with a significant (*p* ≤ 0.05) increase except for the treatment with 50 ppm chem-AgNPs which showed a non-significant (*p* ≤ 0.05) decrease, excluding in the number of leaves which showed a significant (*p* ≤ 0.05) decrease, compared with the control (Table 1). Generally, faba bean growth was induced due to AgNPs treatments in a concentration-dependent manner, i.e., 10 ppm concentration of bio-AgNPs produced prominent effects when compared with the control (Figure 3). Collectively, our results indicate that higher AgNPs (chem- and bio-) concentrations (50 ppm) resulted in weaker growth profiles for faba bean seedlings.

### 2.3. Changes in Photosynthetic Pigments, Total Soluble Sugars, Starch, and Total Carbohydrates Content

As shown in Table 2, variation was observed in the response of the photosynthetic pigments of the leaves of faba bean seedlings primed in 10 and 50 ppm of either AgNO_3_ or AgNPs. AgNO_3_ treatments at a concentration of 10 ppm showed a significant (*p* ≤ 0.05) increase in Chl *a*, and Chl *a* + *b*, while the decrease was significant (*p* ≤ 0.05) for carotenoids and non-significant (*p* ≤ 0.05) for total pigments content, compared with the control. On the other hand, treatment with AgNO_3_ at a concentration of 50 ppm showed a significant (*p* ≤ 0.05) decrease in all photosynthetic pigment fractions (Chl *a*, carotenoids, Chl *a* + *b*, and total pigments), compared with the control. The only exception was that Chl *b* showed no change at either concentration (10 and 50 ppm AgNO_3_) compared with the control value (Table 2). For chem-AgNPs treatments at a concentration of 10 ppm, a significant (*p* ≤ 0.05) increase was recorded in Chl *a*, Chl *b*, Chl *a* + *b*, carotenoids, and total pigments, compared with the control. For chem-AgNPs treatments at a concentration of 50 ppm, a significant (*p* ≤ 0.05) increase was recorded for Chl *a*, Chl *b*, and Chl *a* + *b*, whereas a non-significant (*p* ≤ 0.05) decrease was observed in carotenoids, and total pigments, compared with the control (Table 2). Bio-AgNPs treatments at both concentrations (10 and 50 ppm) showed a significant (*p* ≤ 0.05) increase in Chl *a*, Chl *b*, Chl *a* + b, carotenoids (only with 10 ppm), and total pigments. On the other hand, a non-significant (*p* ≤ 0.05) decrease was evident in carotenoids at 50 ppm for bio-AgNPs only, compared with the control (Table 2). In general, bio-AgNPs treatments, particularly a concentration of 10 ppm, appeared to be superior in enhancing photosynthetic pigment content compared with AgNO_3_ (bulk) or chem-AgNPs. On the other hand, the low concentration of either chem- or bio-AgNPs (10 ppm) was a more effective promoter than the high concentration (50 ppm) for the enhancement of Chl *a,* Chl *b*, Chl *a + b*, carotenoids, and total pigments, whereas 50 ppm chem-AgNPs was the most effective reductor of carotenoids, and total pigments (Table 2). The faba bean seedlings whose seeds were primed in 10 or 50 ppm of either AgNO_3_ or AgNPs (chem- and bio-) showed significant decreases in total soluble sugars, starch, and total carbohydrates of leaves, lower than those of control (Table 2). The magnitude of the decrease was most apparent at the high concentration (50 ppm) of either AgNO_3_ or AgNPs (chem- and bio-). In summary, in AgNPs treatments, the low concentration of either chem- or bio-AgNPs (10 ppm) gave a less negative effect in carbohydrate fractions compared with the high concentration (50 ppm) or AgNO_3_ (bulk) treatments at both concentrations (Table 2).

### 2.4. Changes in the Ultrastructure of Chloroplasts

**Figure 4:**
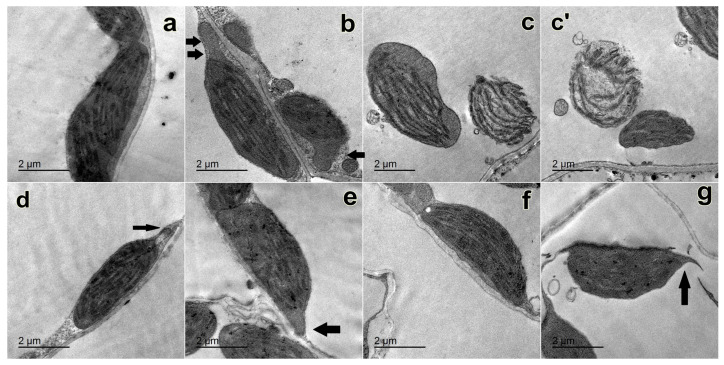
TEM microphotographs of chloroplasts for 21-day-old faba bean leaves with the following treatments: (**a**) control, (**b**) 10 ppm AgNO_3_, (**c**,**c′**) 50 ppm AgNO_3_, (**d**) 10 ppm chemical AgNPs, (**e**) 50 ppm chemical AgNPs, (**f**) 10 ppm biological AgNPs and (**g**) 50 ppm biological AgNPs. Bar = 2 µm. Arrows indicate protrusions extending from chloroplasts.

Careful examination of Figure 4 reveals the direct effect of AgNO_3_ and both types of AgNPs on chloroplast structure. Chloroplasts of the control appeared spindle shaped and were fully stacked with grana (Figure 4a). Chloroplasts of 10 ppm AgNO_3_ appeared with fewer grana and sometimes with oval or spherical shape with extended protrusions (Figure 4b). Meanwhile, the chloroplasts of 50 ppm AgNO_3_ appeared irregular in shape and with degraded envelopes (Figure 4c,c′). Chloroplasts of 10 ppm chem-AgNPs appeared spindle shaped with protrusions (Figure 4d). On the other hand, chloroplasts of 50 ppm chem-AgNPs appeared irregular in shape with fewer grana and with extended protrusions (Figure 4e). Chloroplasts of 10 ppm bio-AgNPs appeared more similar to the control with spindle shape and fully stacked grana (Figure 4f). On the other hand, chloroplasts of 50 ppm bio-AgNPs appeared irregular in shape with fewer grana and with extended protrusions (Figure 4g).

### 2.5. Changes in H_2_O_2_ Content and Antioxidant Enzymes Activities

It is apparent that seed priming with both AgNO_3_ and AgNPs (chem- and bio-) caused a significant increase in H_2_O_2_ content in the leaves of faba bean seedlings compared with the control value (Table 3). The highest value was recorded for AgNO_3_ treatments at concentrations of 50 followed by 10 ppm. On the other hand, AgNPs (chem- and bio-) treatments at concentrations of 10 ppm appeared to produce a significant (*p* ≤ 0.05) increase above the control value compared with the highest concentration (50 ppm) (Table 3).

Antioxidant activities of faba bean seedlings represented by CAT, POX, and PPO concentrations were assayed in the leaves, as tabulated in Table 3. For CAT and POX activities, AgNO_3_ at a concentration of 10 ppm showed a significant (*p* ≤ 0.05) increase, while 50 ppm AgNO_3_ showed a significant (*p* ≤ 0.05) decrease compared with the control value. However, AgNPs (chem- and bio-) treatments at both concentrations (10 and 50 ppm) showed a significant (*p* ≤ 0.05) improvement in the level of CAT and POX, especially in the 10 ppm bio-AgNPs treatment, with the exception of a non-significant (*p* ≤ 0.05) improvement seen with the 50 ppm concentration of chem-AgNPs (Table 3). PPO enzyme behavior was unique, with AgNO_3_ in both concentrations (10 and 50 ppm) recording a significant decrease compared with the control treatment. The reduction rate was more apparent with the high concentration (50 ppm AgNO_3_). For AgNPs (chem- and bio-) treatments, the low concentration (10 ppm) recorded a significant increase whereas 50 ppm recorded a significant decrease, compared with the value of the control. Bio-AgNPs treatments showed superior PPO performance compared with chem-AgNPs in the following sequence: 10 ppm bio-AgNPs > 10 ppm chem-AgNPs > control > 50 ppm bio-AgNPs > 50 ppm chem-AgNPs > 10 ppm AgNO_3_ > 50 ppm AgNO_3_ (Table 3). 

### 2.6. Changes in Silver Content in Shoots and Roots of Faba Bean Seedlings

**Figure 5:**
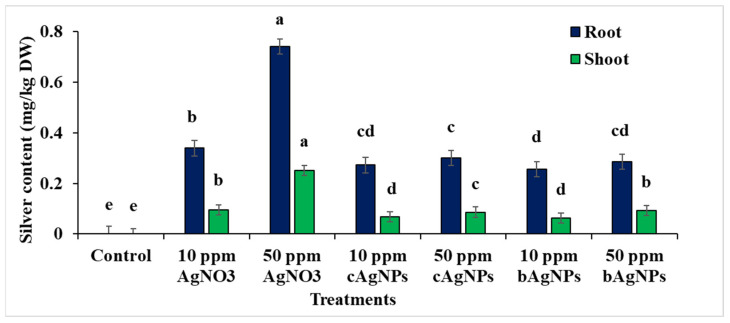
Effects of silver nitrate and silver nanoparticles (chemically or biologically synthesized) on the silver content in roots and shoots of faba bean seedlings. Data with different letters are significantly different (*p* ≤ 0.05). Bars represent standard error (± SE).

The results in Figure 5 show the content of silver in the shoots and roots of 21-day-old faba bean seedlings. It was observed that by increasing the silver nitrate concentration, the content of silver increased in both roots and shoots. However, treatment with both types of AgNPs appeared to be associated with the lowest content of silver in both shoots and roots in a concentration-dependent pattern.

## 3. Discussion

Various physio-chemical approaches are being used nowadays for the synthesis of NPs. However, the progress of efficient green synthesis methods utilizing biological entities with no use of toxic, expensive chemicals and without high energy consumption has attracted researchers to biological methods [32,33]. From the XRD data of both types of AgNPs, the presence of AgCl NPs was apparent in the bio-AgNPs solution with a ratio of Ag:AgCl at 1:2. This means that the important phase in the bio-AgNPs is the AgCl phase. AgCl is a well-known secondary phase commonly produced when using plant-extracts-mediated synthesis [34]. Here, the XRD pattern shows that the synthesis method and conditions highly influence Ag/AgCl secondary phase ratio which was ½ in bio-AgNPs. The particle sizes of both Ag and AgCl phases were approximately similar, which indicated that both types were absorbed by the primed seeds. Devi et al. [35] noted that the interaction between Ag^+^ from AgNO_3_ and Cl from the phytochemical components in the aqueous leaf extract of *O. genistifolia* might initiate AgCl production at room temperature. Phytochemical components in the plant extract acted as reducing agents to convert Ag^+^ ions to metallic Ag during the production of AgCl. The extract’s phenolic compounds’ OH groups reacted with Ag^+^ to generate an intermediate complex, which was then oxidized to decrease Ag^+^ to Ag^o^ NPs. This happened similarly in our investigation, with Cl found in the seed extract of *Jatropha curcas*.

In the present study, priming with AgNO_3_ at concentrations of 10 and 50 ppm induced a variable reduction in all root and shoot vigor parameters, particularly with 50 ppm, compared with the control and AgNPs treatments. Effects of AgNO_3_ were reported to be more toxic than those of AgNPs for *Arabidopsis* seedlings [36] and *A. cepa* roots [37]. This is because of increased ROS formation, which causes oxidative stress [38]. A similar result was reported by Harris [39], in which AgNO_3_ significantly lowered growth metrics by boosting silver absorption in plants. More silver buildup from AgNO_3_ inhibited grain germination, reduced root and shoot length, and decreased chlorophyll pigments in barley seedlings [40]. Our results indicated that priming with chem- and bio-AgNPs at concentrations of 10 and 50 ppm induced root length, and this enhancement was more apparent in bio-AgNPs treatments, particularly at 10 ppm, compared with the control. Similar data were reported by Yan and Chen [41], who noticed that low concentrations of AgNPs had a stimulating effect on the growth of plants. The improvement in plant growth response to AgNPs might be due to the action of ethylene being blocked [42]. Additionally, it was suggested that the effect of AgNPs on the morphological and physiological parameters of plants is related to the morphology of the nanoparticles that are used [42]. However, there are different views about the mechanism of action of silver nanoparticles to trigger a positive effect on plant growth. For example, some researchers suggested that nanoparticles enter the seed coat and have a useful impact on the seed germination processes. Additionally, nanoparticles might induce and increase water absorption by seeds [43]. 

Previous literature highlighted that nano-priming treatment could improve seed water uptake, as primed seeds exhibited faster imbibition in comparison with non-primed ones [44]. Thus, higher water uptake can enhance seed germination and seedling development through complex networks [45]. However, with the exception of 10 ppm bio-AgNPs, all priming concentrations of AgNPs (chem- and bio-) resulted in non-significant reductions in root fresh and dry weights, as well as root water content. The decrease in growth and biomass with increasing concentrations of both types of AgNPs might be attributed to toxicity and the plant cells’ reduced adaptation to AgNPs [46]. For shoot length, 10 ppm bio-AgNPs was the only promoting concentration, while other concentrations of both AgNPs showed variable increases in the other shoot vigor parameters, except for a reduction in dry weight, number of leaves, and total leaf area with the concentration of 50 ppm chem-AgNPs. These findings are consistent with those made by Kaveh [47], who noted a decrease in plant biomass as the concentration of AgNPs increased. The reduction in shoot dry weight, the number of leaves, and the total leaf area of faba bean seedlings may be attributed to a toxic level of NPs [48]. 

For the water content of the roots and shoots of faba bean seedlings, there was a contrast between a decrease and an increase in the AgNPs and AgNO_3_ treatments compared with the control, but the concentration of 10 ppm bio-AgNPs had the greatest positive effect. There could be several reasons for the change in root and shoot water content in faba bean seedlings after priming with nanomaterials. The accumulation of nanomaterials on plant leaves provides a heat source for the canopy, causing changes in gas and moisture exchange due to stomatal blockage, which eventually disrupts various physiological and cellular processes [49]. Furthermore, nanomaterials were shown to change root functionality and hydraulic conductivity by expressing aquaporins and improving osmoregulatory ability in roots, resulting in increased plant water uptake and transport [50]. Moreover, the porosity pattern, as well as the density and size of pores in the root tips of nanocomposite-treated seedlings, may facilitate root water uptake due to decreased root respiration by reducing the living cortical area [51].

Our results indicate that the effect of AgNPs, particularly biosynthesized AgNPs, was apparent on shoot morphological parameters, whereas the effect was non-significant in most of the root parameters. These differences in growth characteristics may be related to the age, method of application, and concentrations of the nanoparticles used [52]. According to Aleksandrowicz-Trzcińska [53], foliar treatment of oak and pine seedlings with a high concentration of AgNPs (50 ppm) resulted in decreased root and shoot growth. Because of their smaller size and relatively large surface area, AgNPs were reported to be more reactive in biological systems than the corresponding bulk metal [54]. The increased biocompatibility of the biogenic AgNPs may be the cause of the faba bean seedlings’ improved growth pattern under bio-AgNPs treatments, especially at low concentrations (10 ppm). According to earlier research, the phytoconstituents in the plant extract may improve biocompatibility, reduce silver ions, and aid in stabilizing and capping nanoparticles [55,56]. Improved seedling growth with biogenic AgNPs has previously been reported for *E. sativa* [57] and Chinese cabbage [58]. Also, it was reported that biosynthesized AgNPs had no significant negative impact on the growth and development of *Bacopa monnieri* seedlings [59,60]. Collectively, the results of the current study show distinct phytostimulatory effects of biogenic AgNPs on both shoot and root growth of faba bean seedlings in comparison with the effects of chemically synthesized AgNPs. These results confirm that the source of the nanoparticles, in addition to their shape and size, may play a major role in determining the impact of the AgNPs. Also, the presence of dangerous chemicals that were adsorbed on the surface of nanoparticles during their synthesis is understood to be the cause of the toxic effects of chemically produced AgNPs [59,60]. From our results, it was observed that silver was found in the roots and shoots of plants treated with both types of AgNPs, indicating the possible toxicity of such AgNPs to the plant. The presence of AgCl in the XRD of the bio-AgNPs could be attributed to the fact that trees of *Jatropha curcas* were irrigated with tap water, and Cl^−^ ions accumulated in the seeds which were used in the biological synthesis of the bio-AgNPs. The corresponding AgCl and AgNPs patterns were deduced from different studies [61,62]. Apparently, the presence of AgCl phase in bio-AgNPs has been neglected in research studies. To our knowledge, all Ag/AgCl NPs solutions were used for medical research not for agricultural use. So, all recorded growth changes could be attributed to both phases in the bio-AgNPs treatments. 

Reduced photosynthetic pigments in exposed plants can be used as a sign of oxidative stress [63]. Priming in AgNO_3_ at a concentration of 10 ppm had a minimally negative effect on the pigment content of faba bean seedling leaves compared with a concentration of 50 ppm, according to our findings. Furthermore, the response of Chl *b* in seedlings primed in both AgNO_3_ concentrations was without change compared with the control. This indicates the negative effects of AgNO_3_ on photosynthetic pigments. This is in accordance with Noori [64], who anticipated that a more stressful environment, such as exposure to a higher concentration of AgNO_3_ or an extended period of exposure, would result in lower photosynthetic pigment content. 

The findings of this study showed that nano-priming in both types of AgNPs resulted in a variable increase in Chl *a*, Chl *b,* Chl *a* + *b*, carotenoids, and total pigments depending on the concentration and source of the nanoparticles. Also, nano-priming with bio-AgNPs was more effective, especially at a 10 ppm concentration. The current study’s findings on the status of Chl *a* which is a biomarker of photosynthetic activity are in line with earlier findings in the literature, which suggest that AgNPs at certain concentrations increase the level of Chl *a* and total chlorophyll in *Brassica juncea* L. because they improve the quantum efficiency of PSII photochemistry in the leaves of treated seedlings [65]. 

According to Giraldo [66], the primary cause of the increased biosynthesis of plastid pigments may be related to faster electron transport rates. As a result of the low concentration (10 ppm) of bio-AgNPs and their positive effects on photosynthetic pigments, this hypothesis could be realized. In the current study, when plants were treated with high doses of bio-AgNPs or chem-AgNPs, the content of the pigments increased at lower AgNPs concentrations. This was consistent with the findings of Khodakovskaya [67], who found that nanomaterials that are toxic at high concentrations may stimulate plant cells at low concentrations. Similar findings were reported for the chlorophyll content of *Chlamydomonas reinhardtii* [68] and *Eichhornia crassipes (Mart) Solms* [69] when those plants were exposed to AgNPs. Increased accumulation of carotenoids and chlorophyll may have a significant impact on the plant’s ability to quench ROS and increase leaf photosynthetic capacity [70].

The changes in the metabolite contents caused by both types of AgNPs and AgNO_3_ treatments were studied by estimating carbohydrate fractions (total soluble sugars, starch, and total carbohydrates) in the leaves of faba bean seedlings. When compared with the control, the contents of these fractions decreased more with AgNO_3_ and AgNPs, especially at high concentrations (50 ppm). According to Singh [32], the sugar content of cauliflower seedlings decreased linearly as NPs concentration increased in comparison with the control treatment. These findings imply that either form of silver may interact with proteins involved in starch synthesis, carbohydrate translocation, or photosystems. Although there are reports that back up this idea that nanoparticles interact with photosystems, the outcomes are inconsistent in nature [59]. Studies of TiO_2_ nanoparticles in *Spinacia oleracea* revealed positive interactions with photosystems, particularly the enhancement of Rubisco activity [41,71], whereas increasing concentrations of aqueous ferrofluid solution revealed an inhibitory effect on *Zea mays* at various stages of photosynthesis via a magnetic influence on the enzymatic structures [72].

When compared with typical chloroplasts of control untreated seedlings of oak and pine, AgNPs caused altered chloroplast ultrastructure, as the chloroplasts appeared lenticular to spherical in form with a high presence of plastoglobules [53]. As a result, it is considered that AgNPs have a negative effect on photosynthetic machinery in some plant species while increasing photosynthetic machinery and stimulating starch accumulation in others as a defence reaction to heavy metal stress produced by AgNPs or AgNO_3_ [53]. These findings appear to be consistent with the negative effects observed in the chloroplast ultrastructure of all treated faba bean seedlings compared with controls (Figure 4). However, nanoparticles had less influence on chloroplast ultrastructure with 10 ppm bio-AgNPs treatments, indicating that lower concentrations of AgNPs are favorable to plants.

Metal nanoparticles significantly affect the ultrastructure of chloroplasts in plants. The magnitude of such changes is clearly proportional to the concentration of the nanoparticle solution. For example, silver nanoparticles at 0.5–3 mg/L modify the morphology of *Arabidopsis* chloroplasts from lens-like to spherical. Simultaneously, the chloroplast stroma becomes more turbid (making it more difficult to distinguish thylakoids inside it), the quantity and size of plastoglobules increases, and thylakoids with inflated lumens appear. Electron-dense inclusions, which the authors refer to as nanoparticle deposits, also appear [73]. The signs of destruction—starch grain formation and a loss of intergranular thylakoids—are visible in duckweed chloroplasts with higher concentrations of silver nanoparticles (5–10 mg/L) [74].

Most of these morphological modifications are well detailed; they are very typical of plants that have been exposed to a variety of severe stimuli at sub-damaging levels. For example, chloroplast rounding is a typical ultrastructural response to a plant’s changed water status and potential osmotic stress [75]. Opacification of the stroma may be caused by changes in its chemical composition [76]. Under osmotic stress, the grana system in the chloroplast is also displaced (typically towards the vacuole) and is commonly explained in terms of the need for fast transport of polyamines into the vacuole to restore cellular homeostasis [77]. The bending of thylakoid membranes is thought to be a marker of oxidative stress damage [76]. Another common adaptive response is the production of chloroplast outgrowths. Under stress, these increase interaction between organelles [78]. Such structural alterations in chloroplasts are frequently observed in response to low or high temperatures, drought, salinization, or exogenous treatments with certain hormones, such as abscisic acid [79,80]. We would like to emphasize that the ultrastructural changes generated by metal nanoparticles in higher plant chloroplasts have received insufficient attention thus far. Nonetheless, the nature of the modifications suggests that the particles trigger structural rearrangements in the plastids directed at adaptations to osmotic and oxidative stressors, which normally occur in the aftermath of nearly any negative impact.

To determine the level of oxidative stress, the amount of H_2_O_2_ in the leaves of faba bean seedlings was measured. In comparison with the control, exposure to silver in the form of AgNO_3_ or AgNPs (chem- and bio-) resulted in significantly higher concentrations of H_2_O_2_ in seedling leaves in a manner dependent on the concentration of the Ag used in priming. Our findings were consistent with those made by Thiruvengadam [81], who examined the effects of exposure to AgNPs in turnip seedlings and discovered that higher AgNPs concentrations resulted in excessive superoxide radical production and increased lipid peroxidation; the formation of H_2_O_2_ was also markedly increased following exposure to 5 and 10 mg/L AgNPs. Purslane [82], pearl millet [83], and wheat [84] plants treated with nanoparticles showed similar effects in terms of increasing the MDA and H_2_O_2_ contents and causing oxidative stress. ROS species, such as H_2_O_2_, act as active regulators of plant development, growth, and physiological responses due to their interaction with different environmental conditions. For this reason, these species are produced in significant concentrations during photosynthesis and respiration [85]. A previous study suggested that H_2_O_2_ is a stress-signaling molecule [86]. Therefore, the results produced by this study could suggest that there is a correlation between the increase in H_2_O_2_ concentration and the reduction in the growth of *Vicia faba* seedlings at the higher AgNPs concentration levels. It is interesting to note that AgNPs produced an increase in H_2_O_2_ concentration, even at a concentration of 10 ppm. Therefore, it can be hypothesized that AgNPs, particularly bio-AgNPs at low concentration levels (10 ppm), can boost plant growth but cause toxicity at higher concentrations (50 ppm).

Changes in the activity of antioxidative enzymes caused by AgNPs point to the occurrence of oxidative stress and ROS action. The results of CAT and POX activities indicated a significant increase with AgNO_3_ at a concentration of 10 ppm, while 50 ppm was associated with a significant decrease compared with AgNPs and the control. On the other hand, PPO activity showed a significant decrease with both concentrations of AgNO_3_, particularly with a concentration of 50 ppm compared with AgNPs and the control. According to Khan [83], *Pennisetum glaucum* L. seedlings displayed decreased antioxidant enzyme activity when exposed to higher concentrations of AgNO_3_ and AgNPs, but the reduction in activity was greater in the case of AgNO_3_. In the current study, seedlings exposed to AgNO_3_ had lower CAT, POX, and PPO activity levels than those exposed to AgNPs. This shows that the ionic form of silver (AgNO_3_) has more phytotoxic effects than nanoparticles. Additionally, another study found that AgNPs elevated ROS in wheat seedlings, which caused plants to experience oxidative stress [87]. 

Environmental stress led to more production of ROS, and plants needed to scavenge ROS for their normal growth, but stress altered the enzymatic activities involved in scavenging ROS [88]. However, in this study, lower CAT, POX, and PPO activity was observed under higher doses of AgNO_3_ and AgNPs, which might be the result of greater ROS production and an alteration in the structure of antioxidants. Our findings are consistent with the findings of Zou [89], who stated that oxidative stress is brought on by AgNPs that interfere with electron transfer. According to earlier reports, the effect of applied AgNPs on antioxidant enzymes varied depending on the plant species, amount, and length of time [90]. The results show that a significant amount of oxidative stress was induced, comparable to rising Ag levels, with altered antioxidative enzyme activity. Overall, it appears that priming *Vica faba* seeds with chemically or biologically prepared AgNPs caused oxidative stress, especially at high concentrations (50 ppm), which was at least partially offset by increased antioxidant enzyme activity, which was more apparent at low concentrations (10 ppm) of bio-AgNPs. These activities appeared to operate together to override unregulated oxidation cascades and protect plant cells from oxidative injury by scavenging ROS.

## 4. Materials and Methods

### 4.1. Materials

A pure strain of *Vicia faba* seeds (cv. Giza 3) was obtained from Sakha Agricultural Research Station, Kafr El-Sheikh, Egypt. Silver nitrate (AgNO_3_) was used as a substrate for the synthesis of AgNPs. AgNO_3_ and trisodium citrate of analytical grade were purchased from local chemical suppliers in Egypt. All the solutions were made using double distilled water.

Preparation of Jatropha seed extract: *Jatropha curcas* seeds were collected from a local source in Egypt. After milling 50 g of seeds (shell + kernel) in an ordinary coffee grinder, the seeds were boiled in 500 mL of double-distilled water for 2 h. A clear seed extract was produced after filtering through Whatman’s No. 1 filter paper and stored at 4 °C in an airtight container for future use [29].

Silver nitrate (bulk) solution preparation: A fresh silver nitrate solution was made by adding 10^−3^ mM silver nitrate to 100 cm^3^ of deionized water (stock solution). 

### 4.2. Synthesis of AgNPs using Trisodium Citrate (Chem-AgNPs)

**Figure 6:**
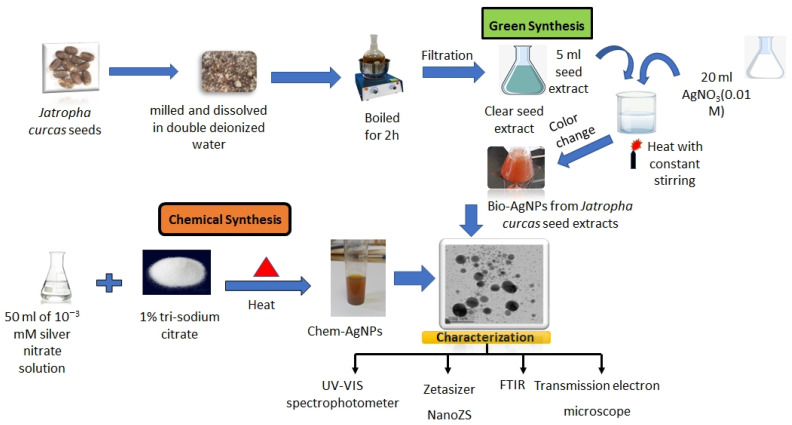
Preparation of silver nanoparticles (chem- and bio-AgNPs).

According to Van Hoonaker [91], the synthesis of silver nanoparticles was achieved by the trisodium citrate chemical reduction method. In the process, 50 cm^3^ of 10^−3^ mM silver nitrate was heated to boiling, then 5 cm^3^ of 1% trisodium citrate was added while stirring until a yellowish brown colour appeared. After that, the mixture was continually stirred to cool to room temperature (25 °C) (Figure 6).

### 4.3. Synthesis of AgNPs using Jatropha Seed Extract (Bio-AgNPs)

In a typical reaction, 20 cm^3^ of 10^−3^ M aqueous silver nitrate solution was added to 5 cm^3^ of seed extract, heated at 80 °C for 15 min, and the resulting solution became crimson. A noticeable SPR band at 430 nm in the UV-VIS spectra served as an indication that silver nanoparticles had been created. The color intensity increased as the concentration of silver nitrate increased at a certain volume fraction (f = 0.2) of seed extract [29] (Figure 6).

### 4.4. Characterization of the Obtained AgNPs (Chem- or Bio-AgNPs)

- Transmission Electron Microscopy (TEM) Analysis of AgNPs

A drop of AgNPs suspension was placed on carbon-coated copper grids to dry at room temperature. The morphology of the AgNPs was determined using a JEOL 1010 transmission electron microscope at 80 kV (JEOL, EM unit, Mansoura University, Mansoura, Egypt).

- Confirming AgNPs Synthesis Using UV-Visible Absorbance Spectroscopy Analysis

The synthesized AgNPs were confirmed by the spectra of the reaction mixture immediately after preparation, using a UV-VIS spectrophotometer (Jenway, UK) at 250 and 700 nm.

- Determination of AgNPs Zeta Potential Analysis

The zeta potential of the AgNPs particles was measured by the determination of the rate at which a particle moved in a known electric field, using a zeta potential analyzer (Malvern Instruments, EM unit, Mansoura University, Mansoura, Egypt). Fourier-Transform Infrared Spectra (FT-IR) of the AgNPs 

FTIR was measured with a PerkinElmer FT-IR in the range of 4000–450 cm^−1^ to identify the functional groups and possible binding sites on adsorbent surfaces. Possible biomolecules responsible for capping and efficient stabilization of the AgNPs were identified.

Finally, characterized chem-AgNPs and bio-AgNPs were used for seed priming application on the *Vicia faba* seeds.

- X-Ray diffraction of the AgNPs

Crystalline metallic silver was examined by X-ray diffraction analysis using an X’Pert PRO PAnalytical-PW 3040/60 X-ray diffractometer with a Cu Kα radiation monochromatic filter in the range 35–80°. The particle size of Ag and AgCl was obtained by the Scherrer equation [92] and quantitative analysis of two phases of Ag/AgCl nanoparticles was also carried out [93].

### 4.5. Experimental Setup and Exposure to AgNPs

*Vicia faba* seeds were sterilized by immersing them in sodium hypochlorite solution (4%) for 3 min, and then properly washed 3 times using distilled water. Thereafter, to break dormancy, seeds were soaked in double-distilled water for 12 h followed by priming separately for 6 h in solutions of either AgNO_3_ (bulk) or AgNPs (chem-AgNPs or bio-AgNPs) according to the following scheme: control (tap water, EC, 0.2 dS/m), 10 ppm AgNO_3_ (bulk), 50 ppm AgNO_3_ (bulk), 10 ppm (chem-AgNPs), 50 ppm (chem-AgNPs), 10 ppm (bio-AgNPs), and 50 ppm (bio-AgNPs). Following the priming period, the seeds were washed thoroughly with distilled water. The sowing of the seeds was done in pots (9 × 7 cm) filled with 250 g sandy clay loam soil (i.e., sand 65.90%, silt 12.40% clay 21.70%, and water holding capacity 3.44%). The soil sample up to a depth of 12 cm was acquired from the horticultural area, Mansoura University, and a detailed analysis of soil physicochemical properties is demonstrated in Table 4. The seeds were divided into 7 groups; each group represented a treatment and contained 20 seeds that were allowed to germinate in 5 plastic pots. Thus, a 5-fold replication in a completely randomized design of 7 treatments was represented. Then, a total of thirty-five pots representing all planned control, AgNO_3,_ and AgNPs treatments were allotted. Seeded pots were positioned in a greenhouse under natural conditions with 76% relative humidity and an average 27/16 °C (day/night) temperature. One week after sowing, thinning was practiced, and three seedlings were retained in each of the treated pots. When required, each pot was supplied with 50 mL of tap water. The treated plants were uprooted carefully after 21 days of growth after sowing. Seedlings were collected and roots and shoots were separated and washed with distilled water for estimation of lengths, fresh and dry weights, water content, number of leaves, and total leaf area. Samples were also taken for the estimation of photosynthetic pigments, total soluble sugars, total carbohydrates, starch, H_2_O_2_, and the activities of some antioxidant enzymes.

### 4.6. Biochemical Analyses

#### 4.6.1. Estimation of Photosynthetic Pigments 

About 0.1g of leaf samples were chopped into little pieces and placed in test tubes with 7 mL of dimethyl sulfoxide (DMSO). The test tubes were placed in a 60°C water bath for 30 min. The tubes were then allowed to cool to ambient temperature before being filtered through Whatman’s No.1 filter paper. DMSO was used to increase the final amount to 10 mL. The absorbance of the extracts was measured at 665.1, 649.1, and 480 nm using a spectrophotometer (JENWAY ST15OSA-Model 7315, Bibby Scientific Ltd., Stone Staffs, UK) against a blank of DMSO. The fractions were approximated as mg g^−1^ fresh weight for the various treatments [94]. 

#### 4.6.2. Evaluation of Total Soluble Sugars, Total Carbohydrates, and Starch 

The anthrone technique [95] was used to assess the concentration of total soluble sugars in the leaf. Two grams of dry leaves were cut, ground to a fine powder, and homogenized in 10 mL of 80% ethanol. The mixture was stirred for 20 min then filtered through Whatman no.1 filter paper and the filterate was collected. The extraction was repeated twice, and the extracts from each sample were mixed. One milliliter of the extract was incubated for 10 min at 90 °C with 5 mL of anthrone solution (0.12 g anthrone in 100 mL 6.5 M H_2_SO_4_). The green product’s absorbance was measured at 630 nm. A standard curve obtained with pure analytical grade glucose was used to calculate glucose equivalents. 

To measure the total carbohydrates, 0.1 g of dry leaf mass was boiled in a water bath for 3 h with 5 mL HCl (2.5 N), then cooled and neutralized with Na_2_CO_3,_ followed by heating 1 mL of the extract in a boiling water bath for 8 min and measuring the cooled samples at 630 nm with a spectrophotometer (JENWAY ST15OSA-Model 7315, Bibby Scientific Ltd., Stone Staffs, UK). The total carbohydrates in plant extracts were calculated using the glucose standard curve [96]. The content of starch was estimated by subtracting total soluble sugars from total carbohydrates.

#### 4.6.3. Measurement of H_2_O_2_ Content and Antioxidant Enzyme Activities

According to Alexieva [97], hydrogen peroxide was measured spectrophotometrically after the reaction with KI. The reaction mixture contained 2 mL of reagent (1 M KI w/v in fresh distilled water), 0.5 mL of 100 mM K-phosphate buffer, and 0.5 mL of 0.1% trichloroacetic acid (TCA) leaf extract supernatant. In the absence of leaf extract, the blank sample had 0.1% TCA. The process was conducted for one hour in complete darkness, and 390 nm absorbance was measured. Using a standard curve created with known values of H_2_O_2_, the amount of hydrogen peroxide was estimated. The antioxidant enzyme activity was measured in extracts made by cold homogenizing 2 g of fresh leaf tissues in 20 mL of 0.1 M phosphate buffer, followed by cold centrifugation at 10,000 rpm for 20 min. Catalase (CAT, EC 1.11.1.6), peroxidase (POX, EC 1.11.1.7), and polyphenol oxidase (PPO, EC 1.10.3.1) were extracted using a pH 6.8 buffer [98]. CAT activity was measured and reported in millimoles of H_2_O_2_ consumed per minute per gram of fresh tissue [99]. POX and PPO activities were then measured in accordance with Devi’s [100] instructions; one enzyme unit was defined.

#### 4.6.4. Determination of Ag in Plant Samples

Approximately 0.2 g dried samples of leaves or root were digested in 10 mL HNO_3_: H_2_O_2_ (1:1, *v*/*v*) at a temperature below 120 °C. After cooling, the mixture was diluted to 10 mL and filtered. The total Ag concentration was determined using atomic absorption spectrophotometry (GBC Scientific Equipment Model SensAA, Dandenong, Victoria, Australia) [101].

### 4.7. Ultrastructure of Chloroplasts Using the Transmission Electron Microscope

To examine the effects of priming seeds in AgNPs on chloroplast structure, the first completely developed leaf of *Vicia faba* seedlings was collected. Transmission electron microscopy (TEM) of leaves was used after they had been cut up and put in a fixative, according to methods from Reynolds [102] and Juniper [103]. A razor blade was used to slice freshly gathered leaves into tiny (1 mm) pieces before exposing them to 2.5% (*v*/*v*) glutaraldehyde. For 24 h, leaf tissues were placed in vials containing 2.5% (*v*/*v*) glutaraldehyde in 1M phosphate buffer at pH 7.5 at 4 °C. Following fixation, the specimens were inserted into gelatin capsules and placed in a 60 °C oven for 60 h. The gelatin capsules were dissolved in boiling water for 1 to 2 h. Using a glass knife, ultra-thin slices were cut on a Reichert ultra-microtome. On the dull surface of form-coated 100- or 200-mesh copper grids, silver or pale gold interference sections were picked up. The grids with sections were dried on clean filter paper, and 2% aqueous uranyl acetate was used to stain ultra-thin slices. A drop of stain was placed in a clean plastic Petri dish, and the grids were gently floated on it, portions facing down. The grids were rinsed with distilled water before being put into drops of lead citrate on a wax plate in a Petri dish. To eliminate carbon dioxide, sodium hydroxide pellets were added to the Petri dish. The grids were immersed in lead citrate for 10 to 20 min before being rinsed with distilled water, dried with a bench lamp, and stored in a grid box. At 80 kV, the stained sections were examined and photographed using a JEOL 1010 transmission electron microscope (EM unit, Mansoura University, Mansoura, Egypt). 

### 4.8. Statistical Analysis

A one-way analysis of variance (ANOVA) with a post hoc Duncan test was used to statistically analyze all the collected data. This analysis was carried out with the Statistical Package for the Social Sciences for Windows (IBM SPSS Statistics, Version 23.0) and statistical significance was defined as a * *p* value ≤ 0.05. 

## 5. Conclusions

**Figure 7:**
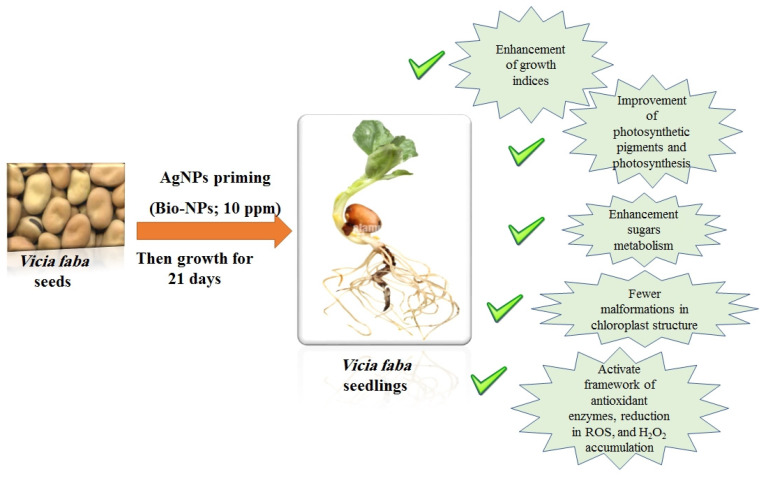
Summary of ameliorative effects of priming seeds in a low dose of AgNPs (Bio-AgNPs; 10 ppm) on the growth of *Vicia faba* seedlings.

Seed priming with AgNPs improved the growth and biomass of faba bean seedlings. The AgNPs enhanced the chlorophyll, carotenoids, starch, and total carbohydrate contents, and the response differed depending on the AgNPs’ concentrations and sources. The chloroplast ultrastructure was less affected by the bio-AgNPs 10 ppm treatment; however, the higher treatments had a detrimental effect, which could be connected to changes in H_2_O_2_ accumulation and antioxidant enzyme activity in faba bean seedling leaves (Figure 7). It is of interest here to state that bio-AgNPs showed much better results than chem-AgNPs and AgNO_3_ treatments. Of interest, the presence of AgCl NPs phase was noted in the bio-AgNPs. To our knowledge, little information has been reported about the effects of Ag/AgCl NPs on plant growth and enzyme activity. We concluded that AgCl produced similar effects to Ag in the bio-AgNPs treatments. In the context of this lack of information, further studies are required to discover the role of AgCl in these bio-AgNPs and what attributes could be connected to this phase. Overall, the seed priming method with lower doses of bio-AgNPs may be employed to boost the growth of faba bean seeds. However, further research is needed to determine the long-term impact of AgNPs on crop productivity in various crop species and the toxic effects of such use.

## Figures and Tables

**Table 1:** Effects of silver nitrate and silver nanoparticles (chemically or biologically synthesized) on growth traits of faba bean seedlings. Data are means ± standard error. Means in each column followed by different letters are significantly different at *p* ≤ 0.05.

Treatments	Root Growth Vigor				Shoot Growth Vigor					
	Root Length (cm/ Seedling)	Root FW (g/Seedling)	Root DW (g/ Seedling)	Root Water Content (g/ Seedling)	Shoot Length (cm/Seedling)	Shoot Fwt (g/ Seedling)	Shoot DW (g/ Seedling)	Shoot Water Content (g/ Seedling)	No. of Leaves/ Seedling	Total Leaf Area (cm^2^/ Seedling)
Control	9.17 ± 0.17 ^bc^	2.54 ± 0.23 ^a^	0.19 ± 0.03 ^b^	2.35 ± 0.22 ^a^	27.67 ± 1.20 ^a^	3.95 ± 0.21 ^ab^	0.31 ± 0.01 ^a^	3.64 ± 0.19 ^ab^	7 ± 0.58 ^b^	0.27 ± 0.03 ^ab^
10 ppm AgNO_3_	8.33 ± 0.60 ^bc^	1.98 ± 0.11 ^b^	0.13 ± 0.03 ^b^	1.85 ± 0.24 ^ab^	20.00 ± 1.15 ^b^	3.13 ± 0.18 ^ab^	0.24 ± 0.04 ^ab^	2.89 ± 0.35 ^b^	6 ± 0.00 ^c^	0.25 ± 0.05 ^b^
50 ppm AgNO_3_	7.17 ± 0.17 ^c^	1.44 ± 0.24 ^b^	0.11 ± 0.01 ^b^	1.33 ± 0.10 ^b^	19.33 ± 0.73 ^b^	2.68 ± 0.39 ^b^	0.19 ± 0.04 ^b^	2.49 ± 0.14 ^b^	5 ± 0.00 ^c^	0.19 ± 0.01 ^b^
10 ppm cAgNPs	10.33 ± 0.83 ^b^	2.31 ± 0.42 ^ab^	0.14 ± 0.05 ^b^	2.17 ± 0.49 ^a^	26.33 ± 2.33 ^a^	4.25 ± 0.21 ^a^	0.35 ± 0.04 ^a^	3.90 ± 0.31 ^ab^	8 ± 0.00 ^a^	0.31 ± 0.04 ^a^
50 ppm cAgNPs	9.50 ± 0.58 ^bc^	2.05 ± 0.55 ^ab^	0.12 ± 0.02 ^b^	1.93 ± 0.40 ^ab^	25.67 ± 1.76 ^a^	3.99 ± 0.73 ^ab^	0.29 ± 0.04 ^ab^	3.70 ± 0.56 ^ab^	6 ± 0.00 ^a^	0.29 ± 0.11 ^ab^
10 ppm bAgNPs	13.33 ± 1.67 ^a^	2.91 ± 0.08 ^a^	0.25 ± 0.09 ^a^	2.66 ± 0.20 ^a^	28.33 ± 0.33 ^a^	4.48 ± 0.35 ^a^	0.36 ± 0.06 ^a^	4.12 ± 0.66 ^a^	8 ± 0.00 ^c^	0.33 ± 0.09 ^a^
50 ppm bAgNPs	11.00 ± 0.58 ^ab^	2.06 ± 0.22 ^ab^	0.13 ± 0.01 ^b^	1.93 ± 0.03 ^ab^	27.67 ± 0.33 ^a^	4.39 ± 0.61 ^a^	0.32 ± 0.02 ^a^	4.07 ± 0.19 ^a^	7 ± 0.00 ^a^	0.31 ± 0.01 ^a^

**Table 2:** Effects of silver nitrate and silver nanoparticles (chemically or biologically synthesized) on photosynthetic pigments (mg/ g FW) and carbohydrates (mg/g DW) of faba bean leaves. Data are means ± standard error. Means in each column followed by different letters are significantly different at *p* ≤ 0.05.

Treatments	Chl *a*	Chl *b*	Car	Chl *a* + *b*	Total Pigments	Total Soluble Sugars	Starch	Total Carbohydrates
Control	0.93 ± 0.07 ^c^	0.13 ± 0.08 ^e^	0.34 ± 0.06 ^c^	1.06 ± 0.05 ^d^	1.40 ± 0.04 ^c^	15.85 ± 0.28 ^a^	260.98 ± 1.83 ^a^	276.83 ± 2.11 ^a^
10 ppm AgNO_3_	0.94 ± 0.06 ^c^	0.13 ± 0.02 ^e^	0.28 ± 0.07 ^e^	1.07 ± 0.04 ^cd^	1.35 ± 0.03 ^c^	8.90 ± 0.21 ^d^	187.56 ± 1.83 ^d^	196.46 ± 2.11 ^d^
50 ppm AgNO_3_	0.84 ± 0.08 ^d^	0.13 ± 0.06 ^e^	0.21 ± 0.07 ^f^	0.97 ± 0.06 ^e^	1.18 ± 0.08 ^d^	6.59 ± 0.49 ^e^	167.93 ± 3.31 ^f^	174.52 ± 3.52 ^f^
10 ppm cAgNPs	1.33 ± 0.16 ^b^	0.29 ± 0.03 ^b^	0.37 ± 0.08 ^b^	1.62 ± 0.09 ^b^	1.99 ± 0.07 ^b^	11.10 ± 0.49 ^c^	198.46 ± 1.83 ^c^	209.56 ± 2.11 ^c^
50 ppm cAgNPs	0.95 ± 0.08 ^c^	0.16 ± 0.09 ^d^	0.28 ± 0.05 ^e^	1.11 ± 0.03 ^c^	1.39 ± 0.06 ^c^	6.61 ± 0.35 ^e^	169.73 ± 0.21 ^f^	176.34 ± 0.70 ^f^
10 ppm bAgNPs	1.56 ± 0.04 ^a^	0.31 ± 0.07 ^a^	0.39 ± 0.07 ^a^	1.87 ± 0.08 ^a^	2.26 ± 0.15 ^a^	13.66 ± 0.28 ^b^	209.88 ± 3.03 ^b^	223.54 ± 3.52 ^b^
50 ppm bAgNPs	1.34 ± 0.17 ^b^	0.28 ± 0.06 ^c^	0.29 ± 0.08 ^d^	1.62 ± 0.04 ^b^	1.91 ± 0.13 ^b^	6.83 ± 0.28 ^e^	173.17 ± 1.76 ^e^	180.00 ± 2.11 ^e^

**Table 3:** Effects of silver nitrate and silver nanoparticles (chemically or biologically synthesized) on hydrogen peroxide and antioxidant enzymes of faba bean leaves. Data are means ± standard error. Means in each column followed by different letters are significantly different at *p* ≤ 0.05.

Treatments	H_2_O_2_ (µmol/g FW)	CAT (mmol H_2_O_2_/min/g FW)	POX (U/min/g FW)	PPO (U/min/g FW)
Control	18.75 ± 0.12 ^g^	5.78 ± 0.02 ^e^	52.92 ± 0.03 ^e^	65.28 ± 0.03 ^c^
10 ppm AgNO_3_	35.35 ± 0.32 ^b^	7.44 ± 0.03 ^d^	76.54 ± 0.05 ^d^	45.83 ± 0.04 ^f^
50 ppm AgNO_3_	58.85 ± 0.45 ^a^	2.51 ± 0.05 ^f^	42.36 ± 0.04 ^f^	29.17 ± 0.02 ^g^
10 ppm cAgNPs	28.35 ± 0.22 ^e^	9.04 ± 0.01 ^b^	85.28 ± 0.06 ^c^	69.17 ± 0.02 ^b^
50 ppm cAgNPs	33.16 ± 0.25 ^c^	7.61 ± 0.03 ^d^	75.14 ± 0.07 ^d^	51.11 ± 0.03 ^e^
10 ppm bAgNPs	21.35 ± 0.12 ^f^	10.09 ± 0.01 ^a^	101.64 ± 0.03 ^a^	77.22 ± 0.04 ^a^
50 ppm bAgNPs	32.45 ± 0.12 ^d^	8.35 ± 0.02 ^c^	88.67 ± 0.11 ^b^	57.50 ± 0.05 ^d^

**Table 4:** Chemical characteristics of the soil used in the current study.

pH	EC (dSm^−1^)	K^+^ (meq/100 g)	Na^+^ (meq/100 g)	Ca^++^ (meq/100 g)	Mg^++^ (meq/100 g)	CO_3_^--^ (meq/100 g)	HCO_3_^−^ (meq/100 g)	Cl^−^ (meq/100 g)
7.97	0.88	0.54	4.26	2.60	0.72	-	2.08	3.37

## Data Availability

All data generated or analyzed during this study are included in this article.

## Supplementary Materials

The following supporting information can be downloaded at: https://www.mdpi.com/article/10.3390/plants12132509/s1, Figure S1: IR spectrum of chemically synthesized silver nanoparticles; Figure S2: IR spectrum of biologically synthesized silver nanoparticles.
