# Supplementary material for: How Can Biological and Chemical Silver Nanoparticles Positively Impact Physio-Chemical and Chloroplast Ultrastructural Characteristics of Vicia faba Seedlings?
Source: Plants (Basel). 2023 Jun 30;12(13):2509. doi: 10.3390/plants12132509 (PMC10347178; doi:10.3390/plants12132509)
Supplement: Supplementary file 1 [file plants-12-02509-s001.zip › plants-2452633-supplementary.pdf]

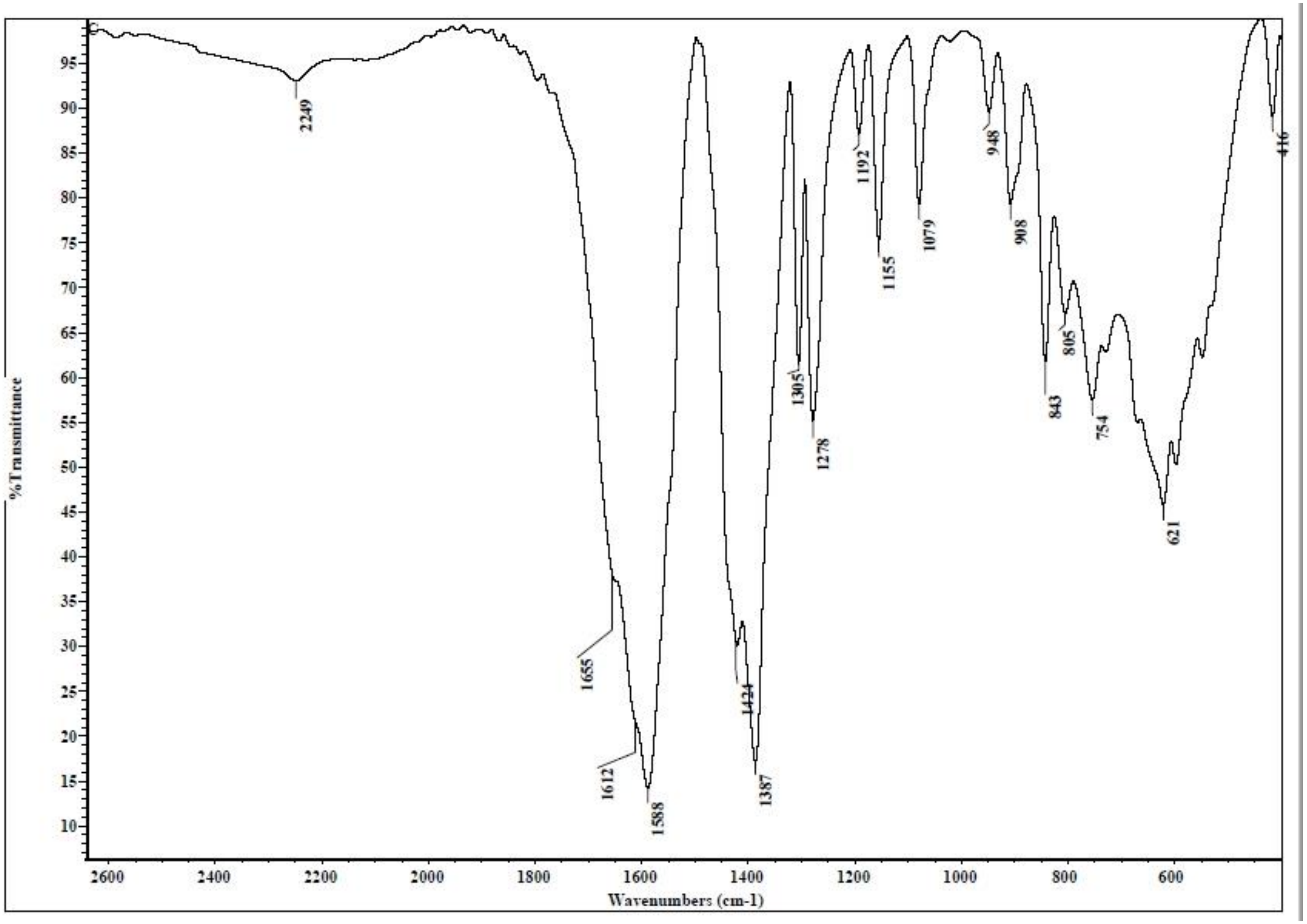


**Supplementary Figure S1.** IR spectrum of chemically synthesized silver nanoparticles.

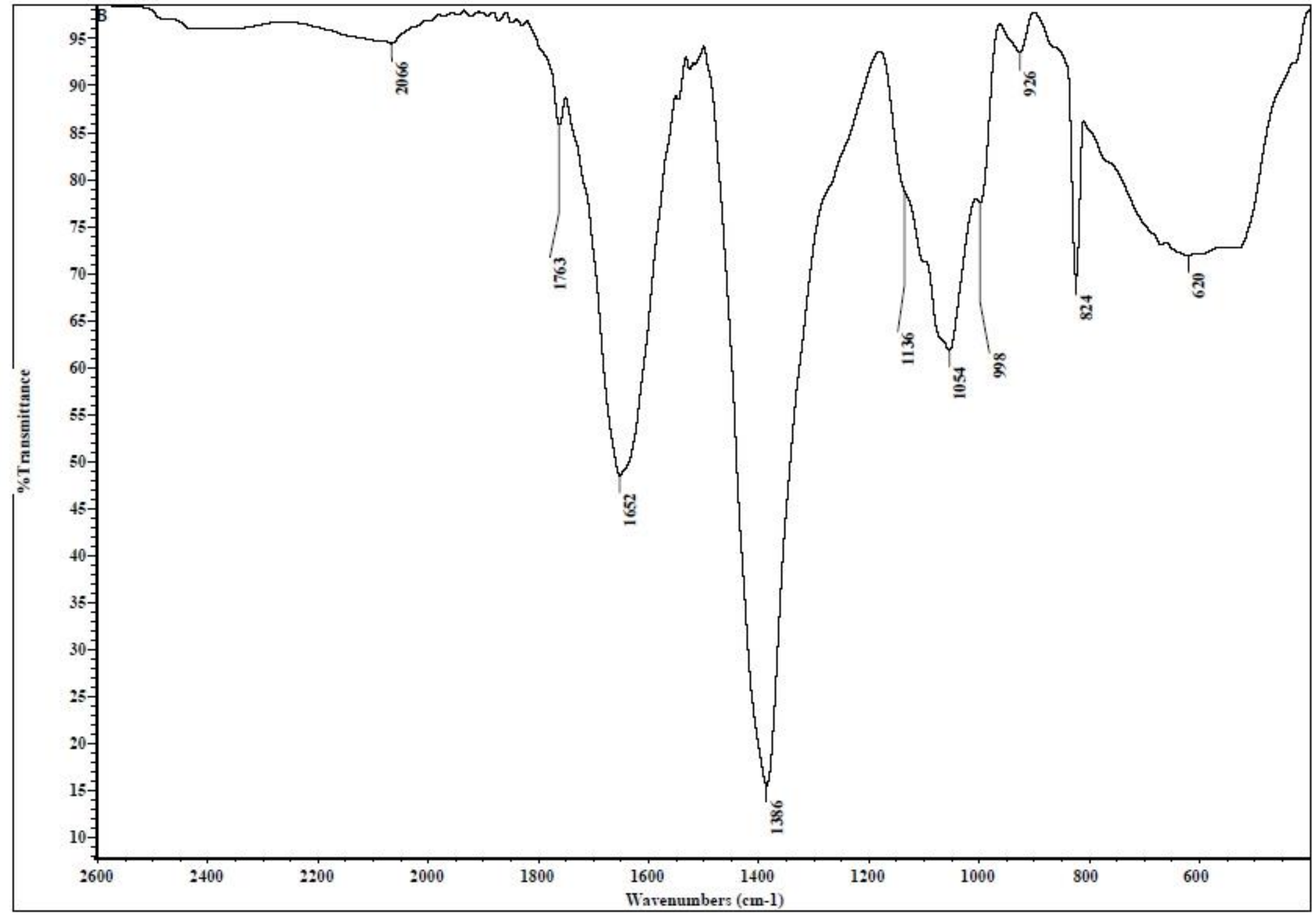


**Supplementary Figure S2.** IR spectrum of biologically synthesized silver nanoparticles.
